# Effective treatment of chemoresistant breast cancer *in vitro* and *in vivo* by a factor VII-targeted photodynamic therapy

**DOI:** 10.1038/bjc.2011.88

**Published:** 2011-03-22

**Authors:** J Duanmu, J Cheng, J Xu, C J Booth, Z Hu

**Affiliations:** 1 Department of Obstetrics, Gynecology and Reproductive Sciences, Yale University School of Medicine, 310 Cedar Street, New Haven, CT 06520, USA; 2 Section of Comparative Medicine, Yale University School of Medicine, New Haven, CT 06520, USA

**Keywords:** ligand-targeted photodynamic therapy, factor VII, tissue factor, chemoresistant breast cancer, neovascular- and cancer cell-targeting photodynamic therapy

## Abstract

**Background:**

The purpose of this study was to test a novel, dual tumour vascular endothelial cell (VEC)- and tumour cell-targeting factor VII-targeted Sn(IV) chlorin e6 photodynamic therapy (fVII-tPDT) by targeting a receptor tissue factor (TF) as an alternative treatment for chemoresistant breast cancer using a multidrug resistant (MDR) breast cancer line MCF-7/MDR.

**Methods:**

The TF expression by the MCF-7/MDR breast cancer cells and tumour VECs in MCF-7/MDR tumours from mice was determined separately by flow cytometry and immunohistochemistry using anti-human or anti-murine TF antibodies. The efficacy of fVII-tPDT was tested *in vitro* and *in vivo* and was compared with non-targeted PDT for treatment of chemoresistant breast cancer. The *in vitro* efficacy was determined by a non-clonogenic assay using crystal violet staining for monolayers, and apoptosis and necrosis were assayed to elucidate the underlying mechanisms. The *in vivo* efficacy of fVII-tPDT was determined in a nude mouse model of subcutaneous MCF-7/MDR tumour xenograft by measuring tumour volume.

**Results:**

To our knowledge, this is the first presentation showing that TF was expressed on tumour VECs in chemoresistant breast tumours from mice. The *in vitro* efficacy of fVII-tPDT was 12-fold stronger than that of ntPDT for MCF-7/MDR cancer cells, and the mechanism of action involved induction of apoptosis and necrosis. Moreover, fVII-tPDT was effective and safe for the treatment of chemoresistant breast tumours in the nude mouse model.

**Conclusions:**

We conclude that fVII-tPDT is effective and safe for the treatment of chemoresistant breast cancer, presumably by simultaneously targeting both the tumour neovasculature and chemoresistant cancer cells. Thus, this dual-targeting fVII-tPDT could also have therapeutic potential for the treatment of other chemoresistant cancers.

Chemotherapy resistance is a major problem in the management of patients with breast cancer. To alternatively and effectively treat chemoresistant breast tumours, we propose to simultaneously target tumour neovasculature and tumour cells using a ligand-targeted photodynamic therapy (PDT) that we recently developed for treatment of breast cancer (Hu *et al*, 2010a, 2010b). Like chemosensitive cancer, chemoresistant cancer requires tumour blood vessels to provide nutrients and oxygen (Bairey *et al*, 2000). It is believed that targeting tumour neovasculature is a better strategy for cancer therapy than targeting cancer cells (Alessi *et al*, 2004), and we believe that targeting both the tumour neovasculature and tumour cells can achieve a better effect than targeting either type of these cells alone (Hu *et al*, 1999, 2010a, 2010b; Hu and Garen, 2000, 2001).

Photodynamic therapy is a treatment involving three components: a photoactivatable photosensitiser (PS), laser light and tissue oxygen. The current applications of PDT involve intravenous injection of PS, followed by irradiation of the diseased lesion with a laser light. The PS can absorb light with a 600–700 nm wavelength. In turn, it converts oxygen to cytotoxic radicals and singlet-oxygen ions. These toxic molecules in the cells then cause cellular necrosis and/or apoptosis. Photodynamic therapy has clinical indications in the treatment of localised cancers and has therapeutic potential for local recurrence of chemoresistant tumours (Capella and Capella, 2003; Merlin *et al*, 2003). However, a serious limitation of the conventional PDT procedure using non-targeted PS (ntPDT) is the toxicity that results from internalisation of the PS by normal cells. To overcome the poor selectivity of PS, antibodies and ligands for receptors on tumour cells were tested to target PS into tumour cells (Mayo *et al*, 2003; Sharman *et al*, 2004). However, there are no published studies to date regarding neovascular- and cancer cell-targeting PDT for chemoresistant breast cancer.

The objective of this study was to target cancer cells and tumour neovasculature for alternative treatment of chemoresistant breast cancer using a factor VII (fVII)-targeted PDT that we recently developed (Hu *et al*, 2010a, 2010b). It is known that fVII is a natural ligand for receptor tissue factor (TF), with high specificity and affinity (dissociation constant up to 1 pM) (Waxman *et al*, 1992). The TF expression is detected on endothelial cells of pathological capillary blood vessels associated with solid tumours (Contrino *et al*, 1996; Shoji *et al*, 1998; Hu *et al*, 1999; Hu and Garen, 2001; Tang *et al*, 2007), wet macular degeneration (wMD) (Bora *et al*, 2003; Tezel *et al*, 2007) and endometriosis (Krikun *et al*, 2010); however, TF is not expressed on endothelial cells of normal blood vessels (Drake *et al*, 1989; Flossel *et al*, 1994; Contrino *et al*, 1996; Hu *et al*, 1999; Hu and Garen, 2001). In addition, TF is overexpressed in cancer cells and cancer stem cells in solid tumours (Callander *et al*, 1992; Shoji *et al*, 1998; Hu *et al*, 1999; Hu and Garen, 2000, 2001; Fernandez and Rickles, 2002; Forster *et al*, 2006; Milsom *et al*, 2007) and leukaemia (Andoh *et al*, 1987; Bauer *et al*, 1989; Hair *et al*, 1996). Therefore, TF can be regarded as a common but specific therapeutic target on angiogenic tumour vascular endothelial cells (VECs) and tumour cells based on its selective expression. Using a well-established multidrug resistant (MDR) breast cancer MCF-7/MDR line (Kiss *et al*, 1994) as a model resistant cancer line, we report for the first time that TF expression is observed on tumour VECs in chemoresistant human breast tumour xenograft in mice and then report the efficacy and safety of fVII-targeted SnCe6 PDT (Hu *et al*, 2010a) for effective treatment of chemoresistant breast cancer *in vitro* and *in vivo*.

## Materials and methods

### Cell lines

Chinese hamster ovary cells (CHO-K1, ATCC) were grown in F-12 medium. The human breast cancer parental chemosensitive MCF-7 and chemoresistant MCF-7/MDR (kindly provided by Dr Zping Lin and Dr Alan Clayton Sartorelli at Yale University) were grown in DMEM supplemented with 10% FBS and 1 : 100 penicillin/streptomycin (Sigma, St Louis, MO, USA) at 37 °C and 5% CO_2_.

### Western blotting for MDR (gp170) expression by MCF-7/MDR

Human breast cancer MCF-7 and MCF-7/MDR cells were grown in growth medium in six-well plates. When the cells reached ∼90% confluence, the cells were washed once with PBS and cell membrane proteins were extracted with M-PER mammalian membrane extraction solution (Pierce, Rockford, IL, USA) and subjected to SDS–PAGE and western blotting analysis. For the western analysis, the nitrocellulose membrane (NC) was first probed with 1 *μ*g ml^–1^ mouse anti-gp170 (clone C219 from Covance, Princeton, NJ, USA), followed by 0.01 *μ*g ml^–1^ anti-mouse IgG HRP (Vector Laboratories, Burlingame, CA, USA) and ECL reagents (GE Healthcare, Piscataway, NJ, USA). After exposure to gp170, the NC membrane was stripped using stripping buffer (Pierce), followed by incubation with 1 *μ*g ml^–1^ anti-GAPDH monoclonal antibody (Research Diagnostics Inc., Concord, MA, USA), 0.01 *μ*g ml^–1^ anti-mouse IgG HRP and ECL to ensure equal loading of protein samples for the western analysis.

### Flow cytometry for TF expression using monoclonal anti-HTF

Flow cytometry was performed using 20 *μ*g ml^–1^ goat anti-HTF (American Diagnostica, Stamford, CT, USA), followed by 20 *μ*g ml^–1^ secondary anti-goat IgG Fc FITC (Vector Laboratories) similar to previously described methods (Hu *et al*, 1999, 2010a; Hu and Garen, 2000, 2001).

### Production and purification of the mfVII protein

The plasmid containing mfVII cDNA was constructed, as described in our recent papers (Hu *et al*, 2010a, 2010b). Briefly, the mouse factor VII cDNA containing a K341A mutation was derived from a previously constructed Icon plasmid vector pcDNA3.1(+)/mfVII(K341A)/hIgG1 Fc (Hu *et al*, 1999; Hu and Garen, 2000, 2001). The new mfVII cDNA was composed of the coding sequence of mfVII, a *Bam*HI site, and a sequence encoding the first 15 amino-acid residues of ribonuclease *S*-peptide with a D14N mutation (Kim and Raines, 1993) followed by eight histidines (His tag) (mfVII(K341A)/Sp(D14N)/His, abbreviated as fVII, unless specified). The *S*-peptide and His tag were designed for detection and purification of fVII proteins. CHO-K1 cells were stably transfected with the plasmid vector and grown in serum-free medium SHM4CHO (Thermo Scientific, Basingstoke, UK) supplemented with a final concentration of 1 *μ*g ml^–1^ vitamin K1 (Sigma) similar to previously described methods (Hu *et al*, 1999; Hu and Garen, 2000, 2001). The fVII protein as a secreted protein was purified from the collected SFM from the CHO-K1 cells using Ni-NTA affinity resins for His-tagged proteins (Qiagen, Valencia, CA, USA), as described (Hu *et al*, 2010a, 2010b).

### Conjugation of fVII protein to SnCe6

The following conjugation procedure has been used to conjugate COOH group-containing photosensitisers (verteporfin and SnCe6) to fVII protein using crosslinker EDC (*N*'-3-dimethylaminopropyl-*N*-ethylcarbodiimide hydrochloride; Sigma), as described in detail by Hu *et al* (2010a, 2010b). Briefly, Sn(IV) Ce6 dye (MW 850.21) (Frontier Scientific, Logan, UT, USA) was first activated by EDC, and then fVII protein was added to the activated dye for conjugation. The fVII-conjugated SnCe6 was separated from unconjugated SnCe6 using Sephadex G50 spin columns (Roche, Indianapolis, IN, USA). The collected fVII-SnCe6 conjugate was characterised by spectral scanning (200–800 nm) and by reading absorbance at 635 nm (A635 nm) using a spectrophotometer (Beckman, Brea, CA, USA). The protein concentration was determined by Protein Assay Reagent (Bio-Rad, Hercules, CA, USA), and the concentration of SnCe6 was calculated based on A635 nm using a SnCe6 standard curve. Based on their molar concentrations (*μ*M), the molar ratio of dye to protein was (4.21±0.95) : 1 (mean±s.d., *n*=11), as the same preparations of the fVII-SnCe6 conjugates as reported in our recent article (Hu *et al*, 2010a) were used in this study.

### *In vitro* PDT in tissue culture plates

The *in vitro* PDT tests were done in 96-well plates containing 1 × 10^4^ cells in 100 *μ*l growth medium per well, as described in the supplementary information in Hu *et al* (2010a). Briefly, fVII-tPDT or ntPDT was carried out by incubating the cells with different concentrations of fVII-SnCe6 conjugate or unconjugated SnCe6 at 37 °C for 90 min, followed by washing the cells once and adding the growth medium. The cells were irradiated with a 635 nm fibre-coupled diode laser (BWF2-635-0.1-100-0.22; B&W Tek Inc., Newark, DE, USA) for various time durations at 100 mW cm^–2^. Controls included an untreated cell control and a maximal killing control. All of the controls included the same number of cancer cells. In the maximal killing control wells, cells were lysed by addition of 1/10 volume of 9% Triton X-100 45 min before crystal violet staining. After treatment with PDT, the cells were incubated at 37 °C and 5% CO_2_ until the untreated cells in the control wells reached 95% confluence and then the cells in the experimental and control wells were fixed and stained for the crystal violet staining assay described below.

### Crystal violet staining to determine the *in vitro* efficacy of SnCe6 PDT

Non-clonogenic crystal violet staining for monolayer cell membrane loss was performed as previously described (Mickuviene *et al*, 2004) with a minor modification for the more convenient reading of absorbance at 595 nm (A595 nm) (Hu *et al*, 2010a, 2010b). The *in vitro* efficacy was presented as the percentage of surviving cells based on A595 nm readings by the crystal violet staining assay. *Percent of surviving cells* (%)=*(A595 nm of PDT-treated cells*−*average A595 nm of maximal killing controls)/(average A595 nm of untreated controls*−*average A595 nm of maximal killing controls)* × *100*%.

### Assessing apoptosis and necrosis as a mechanism of action of SnCe6 PDT

MCF-7/MDR breast cancer cells were treated in 96-well plates with fVII-tPDT or ntPDT (2 *μ*M, 72 J cm^–2^) as described above. At various time points after PDT treatment, the plates were separately assayed for necrosis (cytotoxicity) using the fluorescence-based Cytotox-ONE Homogeneous Membrane Integrity Assay kit (Promega) and apoptosis using the Apo-ONE Homogeneous Caspase-3/7 Assay kit (Promega, Madison, WI, USA). This was performed by measuring fluorescence on a fluorescence microplate reader (SpectraMax Gemini XS, Molecular Devices, Sunnyvale, CA, USA) following the manufacturer's instructions*. Percent of cytotoxicity or apoptosis*=*(fluorescence in treated cells–fluorescence in untreated cells)/(fluorescence in maximal killing control–fluorescence in untreated cells) × 100*%.

To visualise the early events of apoptosis and necrosis, 2 × 10^4^ MCF-7/MDR cancer cells per well were seeded in 96-well plate overnight. Next day, the cells were separately treated by fVII-tPDT using fVII-SnCe6 conjugate and ntPDT using unconjugated SnCe6 (2 *μ*M SnCe6 and 36 J cm^–2^), or were not treated as untreated control. Immediately after the treatment the cancer cells were stained with 1 : 20 diluted Annexin V-FITC for early detection of apoptotic cells and then stained with 1 *μ*g ml^–1^ propidium iodide (PI) for necrotic cells using ApoDETECT ANNEXIN V-FITC KIT (Invitrogen, Carlsbad, CA, USA). After staining, the cells were observed and photographed under fluorescence microscope.

### *In vivo* fVII-tPDT treatment of human chemoresistant breast cancer MCF-7/MDR tumour xenografts in a nude mouse model

The animal study protocol was reviewed and approved by the Institutional Animal Care and Use Committee of Yale University. Human chemoresistant tumour xenografts were generated by subcutaneous injection of 150 *μ*l equal volume mixture of PBS and Matrigel (BD Biosciences, Bedford, MA, USA) containing 3 × 10^6^ MCF-7/MDR cancer cells per mouse in 4- to 6-week-old female athymic nude mice (NCr nude homozygous, Taconic Farms, Germantown, NY, USA) s.c. implanted with 17 *β*-estradiol pellet (60 day release, 1.7 mg per pellet) (Innovative Research of America, Sarasota, FL, USA), as described (Hu *et al*, 1999, 2010a, 2010b; Hu and Garen, 2000, 2001). After the tumours reached 100–200 mm^3^ in size, fVII-SnCe6 conjugate was intravenously (i.v.) injected into mouse tail veins at a final concentration of 2 *μ*M SnCe6 using an estimate of blood volume based on body weight (35 ml blood per kg of mouse body weight) (Hu *et al*, 2010a, 2010b). At 90 min following the injection of fVII-SnCe6, the tumours were irradiated with 65 J cm^–2^ using a 635 nm laser under anaesthesia by intraperitoneal (i.p.) injection of 100 mg ketamine per kg+10 mg xylazine per kg. The date for the first treatment was designated as day 0. The fVII-tPDT treatment procedure, that is, systemic injection of fVII-SnCe6 conjugate and 90-min post-irradiation of the tumour with 635 nm laser light, was repeated on days 6, 12, 20 and 27. The control mice were i.v. injected with only saline buffer.

Note that we did not test the ntPDT control using unconjugated SnCe6 in this animal study. The reason is as follows. (1) We showed in the Results section below that the projected half-maximal effective concentration (EC_50_) of laser fluence in ntPDT using 2 *μ*M unconjugated SnCe6 was 5571.6 J cm^–2^ *in vitro* in tissue culture plates, whereas the EC_50_ of laser fluence in fVII-tPDT (2 *μ*M SnCe6 in fVII-SnCe6 conjugate) was 300-fold less (18.3 J cm^–2^; Figure 2C). Therefore, we anticipate that ntPDT at 2 *μ*M SnCe6 and 65 J cm^–2^ would not have any effect on inhibiting the tumour growth *in vivo* in mice. (2) We carried out four animal experiments in our recent studies on fVII-tPDT for treatment of chemosensitive breast cancer, and three of the ntPDTs using fVII-verteporfin or fVII-SnCe6 conjugate were tested and compared with fVII-tPDT and we did not observe any effect of ntPDT on inhibiting the tumour growth (Hu *et al*, 2010a, 2010b).

The efficacy of fVII-tPDT was determined by measuring tumour width and length with calipers and calculating tumour volume (mm^3^) using the formula (width)^2^ × length/2 as previously described (Hu *et al*, 1999; Hu and Garen, 2000, 2001). At the end of the experiments, the mice were examined morphologically, tumour tissues were fixed in zinc fixative (BD Biosciences), processed, paraffin embedded, sectioned and stained by haematoxylin and eosin (HE) by routine methods. Tumour slides were examined blind to experimental manipulation (by CJB). Unstained tissue sections were used for immunohistochemistry below. Blood samples from the mice were examined for complete blood counting and differential analysis as additional safety assays by Antech Diagnostics (Lake Success, NY, USA).

### Immunohistochemistry (IHC) using rabbit polyclonal antibody to murine TF for staining TF on VECs in MCF-7/MDR tumours from mice

Based on the peptide sequence described in a previous paper (Dackiw *et al*, 1996), we had Sigma-Genosis (The Woodlands, TX, USA) produce antisera to the same 15 amino-acid residues (CITYRKGSSTGKKTN) at the N-terminus murine TF (MTF-N) using keyhole limpet haemocyanin-peptide to immunise New Zealand white rabbits. The polyclonal antibody was affinity purified from the antisera using an MTF-N peptide-coupled Sepharose 4B column and was characterised by SDS–PAGE, dot blotting and flow cytometry. We confirmed that it could bind to murine TF and had crossreaction with human TF but with less binding activity than anti-HTF antibody (HTF1) (Hu *et al*, unpublished data).

Immunohistochemistry was performed as follows. MCF-7/MDR tumour issue slides from control mice were prepared as described above and were deparaffinised in xylene and rehydrated through graded ethanol to distilled water and microwave-heated in an antigen unmasking solution (Vector Laboratories) for antigen retrieval. The sections were then incubated with 3% H_2_O_2_ for 5 min and blocked with 10% goat serum. The sections were separately incubated with 10 *μ*g ml^–1^ rabbit anti-MTF-N for TF staining and with rabbit anti-von Willebrand factor (vWF, factor VIII-related antigen, crossreaction with human, mouse and rat origins) (Millipore/Chemicon, Billerica, MA, USA) for endothelial staining at 37 °C for 1 h. Controls were incubated with isotype rabbit IgG or with PBS buffer. Finally, the sections were incubated using 2 *μ*g ml^–1^ of a secondary anti-rabbit IgG alkaline phosphatase (Vector Laboratories) for 1 h at 37 °C. Positive blue staining for VECs and MTF was visualised by Vector Blue substrate and cell nuclei (red) were counterstained by Vector Nuclear Fast Red (Vector Laboratories).

### Statistical analyses

*In vitro* studies were carried out in duplicate wells for each group, and *in vivo* efficacy in mouse tumour models were assessed in groups of five mice per group. These data are presented as mean±s.d. The statistical significance of differences between the treated and control groups were analysed using paired or unpaired *t*-tests or one-way ANOVA with Tukey's multiple comparison test or two-way ANOVA tests using Prism 5.0c (GraphPad Software, Inc., La Jolla, CA, USA). The *P-*values of <0.05 were considered to be statistically significant. The half-maximal effective concentrations of fVII-tPDT and ntPDT were calculated by the best-fit linear regression or nonlinear one-phase decay equations using Prism 5.0c.

## Results

### TF is expressed by chemoresistant breast cancer cells and tumour VECs in MCF-7/MDR tumours from mice

**Figure 1:**
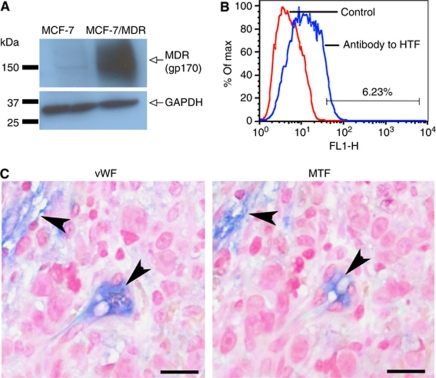
Tissue factor (TF) expression by chemoresistant breast cancer MCF-7/MDR cells and VECs in MCF-7/MDR tumours. (**A**) Multidrug resistance (MDR; gp170) expression by western blotting. Glyceraldehyde 3-phosphate dehydrogenase (GAPDH) was used for comparison of protein loading. (**B**) Tissue factor expression on MCF-7/MDR cancer cells was 6.23% by flow cytometry using 20 *μ*g ml^–1^ goat anti-HTF (blue line). The control was the nonspecific secondary antibody FITC (red line). (**C**) Tissue factor expression (blue) by VECs (MTF, arrowheads) in MCF-7/MDR tumours by immunohistochemistry using a rabbit polyclonal antibody to MTF-N. The endothelial origin of these VECs was confirmed by positively staining (blue) for the endothelial marker vWF (vWF, arrowheads). Vector Blue chromogen and Nuclear Fast Red counterstain. Scale bar=20 *μ*m.

We first confirmed the expression of MDR (P-glycoprotein 170, gp170) by the chemoresistant MCF-7/MDR breast cancer line using western blotting with anti-gp170 antibody. Expression of gp170 was indeed detected in the cell membrane protein extracts from MCF-7/MDR cells but not from MCF-7 cells (Figure 1A).

We next determined the expression of TF on MCF-7/MDR cancer cells and on VECs in MCF-7/MDR tumour xenografts in nude mice. Using flow cytometry with goat anti-human TF antibody, we showed that MCF-7/MDR cancer cells expressed TF with an expression level of 6.23% (Figure 1B), similar to the level (8.16%) on the parental chemosensitive MCF-7 line (Hu *et al*, 2010a). Using immunohistochemical staining with a polyclonal antibody to mouse TF (MTF), we showed that TF was expressed by the VECs of capillary vessels with only one thin endothelial layer in MCF-7/MDR tumours (Figure 1C, MTF, arrowheads). The endothelial origin of these MTF-positive cells was confirmed by positive staining for vWF (Figure 1C, vWF, arrowheads), which labels endothelial cells as marker better than CD31 (Bairey *et al*, 2000). Note that isotype rabbit IgG with secondary antibody alkaline phosphatase conjugate or secondary antibody conjugate alone did not have any positive blue staining (not shown). Thus, we conclude that TF is expressed on both the cancer cells and tumour VECs in chemoresistant MCF-7/MDR breast tumours.

### fVII targeting enhances the effect of SnCe6 PDT

**Figure 2:**
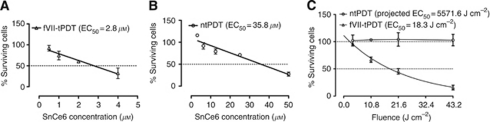
Factor VII (fVII) targeting enhances the effect of SnCe6 PDT, and fVII-tPDT is effective in eradicating MCF-7/MDR cancer cells. (**A** and **B**) The EC_50_ of SnCe6 was 2.8 and 35.8 *μ*M in fVII-tPDT and ntPDT (18 J cm^–2^, laser fluence) for MCF-7/MDR cells, respectively. (**C**) MCF-7/MDR cells had a laser fluence-dependent response to fVII-tPDT (2 *μ*M SnCe6) but had no response to ntPDT (2 *μ*M SnCe6).

To assess whether fVII targeting enhances the effect of SnCe6 PDT, we compared the EC_50_ of SnCe6 as an indicator for the *in vitro* efficacy of fVII-tPDT and ntPDT (laser fluence 18 J cm^–2^) for MCF-7/MDR cancer cells. As shown in Figure 2A, the EC_50_ of SnCe6 in fVII-tPDT was 2.8 *μ*M, whereas it was 35.8 *μ*M in ntPDT (Figure 2B). These results suggest that fVII targeting enhances the efficacy of ntPDT for killing of MCF-7/MDR cancer cells by 12.8-fold.

To further confirm the enhanced efficacy, we treated the MCF-7/MDR cells with fVII-tPDT and ntPDT with 2 *μ*M SnCe6 and varied the laser fluence. We found that the effect of fVII-tPDT (2 *μ*M SnCe6) was laser fluence dependent with an EC_50_ of fluence at 18.3 J cm^–2^ for MCF-7/MDR cancer cells, whereas ntPDT at the same SnCe6 concentration had no effect on killing the cancer cells even at 43.2 J cm^–2^ and its projected EC_50_ was 5571.6 J cm^–2^ (Figure 2C), >300-fold than that in fVII-tPDT (18.3 J cm^–2^). These results are consistent with the results in Figure 1A (i.e., the EC_50_ of SnCe6 was 2.8 *μ*M when fluence was 18 J cm^–2^).

**Figure 3:**
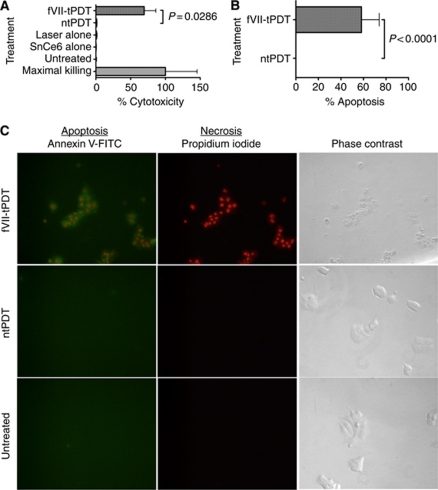
Factor VII-targeted photodynamic therapy (fVII-tPDT) induces significantly stronger levels of apoptosis and necrosis in MCF-7/MDR cancer cells compared with ntPDT. Necrosis (cytotoxicity) (**A**) and apoptosis (**B**) were detected in fVII-tPDT (2 *μ*M SnCe6 and 36 J cm^–2^)-treated MCF-7/MDR cells. (**C**) Apoptosis and necrosis were visualised by immunofluorescence staining in fVII-tPDT (2 *μ*M SnCe6 and 36 J cm^–2^)-treated MCF-7/MDR cells. Note that the fVII-tPDT-treated MCF-7/MDR cells were positively stained for necrosis (red in the nuclei) and apoptosis (green), whereas the ntPDT using unconjugated SnCe6 (2 *μ*M, 36 J cm^–2^)-treated cells and the untreated control cells did not show any staining for apoptosis and necrosis. Note that the fVII-tPDT-treated MCF-7/MDR cells were clearly damaged, whereas the ntPDT-treated and untreated control cells were intact (phase contrast). Original magnification × 400.

From the same experiments as in Figure 2C, we photographed the cancer cells before they were fixed for crystal violet staining. The fVII-tPDT-treated MCF-7/MDR cancer cells became debris even by laser fluence at 10.8 J cm^–2^, and none of the cancer cells remained intact after being treated with 43.2 J cm^–2^ fVII-tPDT (data not shown). In contrast, no cellular damage was observed in the untreated cells or any of the ntPDT-treated cells, even by the highest fluence (43.2 J cm^–2^; data not shown). Similar observations on cellular morphology are shown in Figure 3 below. We conclude that fVII targeting enhances the effect of SnCe6 PDT, and fVII-tPDT is effective in eradicating MCF-7/MDR cancer cells *in vitro*.

### fVII-tPDT induces apoptosis and necrosis as mechanisms of action

To understand the mechanisms of action of fVII-tPDT, we assessed the ability of fVII-tPDT to induce apoptosis and necrosis in MCF-7/MDR cancer cells 1 h after treatment *in vitro*. Figures 3A and B show that fVII-tPDT (2 *μ*M and 36 J cm^–2^) induced significantly higher levels of necrosis (Figure 3A) and apoptosis (Figure 3B) than ntPDT (*P*=0.0286 and *P*<0.0001 by one-way ANOVA, respectively), whereas laser alone and SnCe6 alone induced traces of necrosis, similar to that in ntPDT-treated cells (*P*=0.84 and *P*=0.27 *vs* ntPDT by one-way ANOVA, respectively; Figure 3A).

When visualising early necrosis and apoptosis, we observed that all the fVII-tPDT-treated MCF-7/MDR cells were stained clearly for obvious apoptosis (green) and necrosis (red) (Figure 3C), whereas the ntPDT-treated and the untreated control cells were not stained for apoptosis and necrosis (Figure 3C), consistent with the results in Figures 3A and B. Thus, we conclude that fVII-tPDT induces significant apoptosis and necrosis in chemoresistant breast cancer cells immediately after the treatment when compared with ntPDT.

### fVII-tPDT is effective and safe for treatment of chemoresistant MCF7/MDR tumours in a nude mouse model

**Figure 4:**
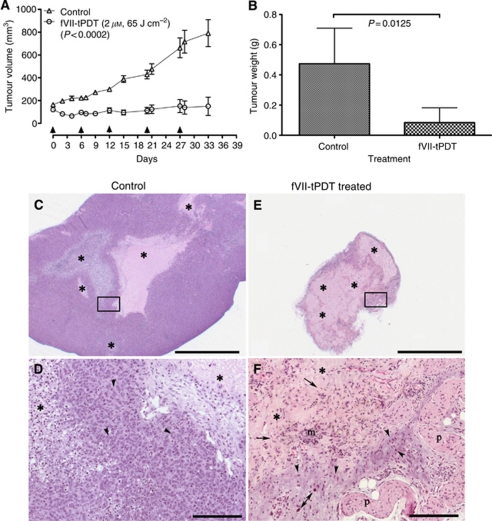
Factor VII-targeted photodynamic therapy (fVII-tPDT) is effective in the treatment of chemoresistant breast cancer MCF-7/MDR tumours in nude mice. (**A** and **B**) After fVII-tPDT treatment (2 *μ*M, 65 J cm^–2^; arrows indicating the treatment dates), MCF-7/MDR tumour volume (**A**) and tumour weight (**B**) from fVII-tPDT-treated mice were significantly smaller than those from control mice. (**C**–**F**) Histopathology of MCF-7/MDR tumours from control and fVII-tPDT-treated mice. MCF-7/MDR tumours from control mice (**C** and **D**) were large and had multiple variably sized foci of necrosis and cellular debris (^*^) in contrast to the tumours from fVII-tPDT-treated mice (**E** and **F**) that were smaller and composed predominantly of necrotic cellular debris (^*^). At increased magnification, tumour cells from MCF-7/MDR tumours (**D**, inset of **C**) were numerous, easy to identify by their dark blue euchromatic nuclei. In contrast, distinct tumour cells from fVII-tPDT-treated (**F**, inset of **E**) mice were markedly fewer in number and in the process of necrosis or apoptosis as evidenced by the diffuse nuclear pkynosis (arrowheads), including the nuclei of large tumour cells (double arrow). Tumour cells were enmeshed in the background of necrotic cellular debris (^*^) admixed with haemosiderin-laden macrophages (m) and fibroblasts (arrows). Haematoxylin and eosin (HE), scale bars for (**C** and **E**)=2000 *μ*m; for (**D** and **F**)=200 *μ*m. p=panniculus carnosus muscle.

To assess the *in vivo* efficacy and safety of fVII-tPDT for chemoresistant breast tumours, we first generated subcutaneous MCF-7/MDR tumour xenografts in nude mice. After tumours formed with sizes of ∼100–200 mm^3^, the mice (*n*=5) were treated by fVII-tPDT (2 *μ*M SnCe6 and 65 J cm^–2^) on days 0, 6, 12, 20 and 27. The control mice (*n*=5) were i.v. injected with only PBS buffer. The tumour growth was significantly inhibited by fVII-tPDT compared with control mice (Figure 4A; *P*=0.0002 by two-tailed *t*-test). The fVII-tPDT-treated tumour weights were also significantly lighter than control tumours (Figure 4B; *P*=0.0125 by two-tailed *t*-test).

In addition, the histopathology of stained MCF-7/MDR tumours from control mice revealed large tumours comprising predominantly tumour cells (Figure 4A) with multiple variably sided foci of necrosis (Figures 4C and D). In contrast, fVII-tPDT-treated tumours were small and comprised predominantly necrotic cellular debris (Figure 4E) with markedly fewer tumour cells and in the process of necrosis or apoptosis as evidenced by the diffuse nuclear pkynosis (Figure 4F), including the nuclei of large tumour cells. The tumour cells were surrounded by necrotic cellular debris, with frequent foci of haemosiderin-laden macrophages and fibroblasts (Figure 4F).

Regarding the safety, we did not observe any obvious morphological side effects in the fVII-tPDT-treated mice during the experiments and at the time of killing the mice. In addition, there was no statistical difference between control mice and fVII-tPDT-treated mice in terms of complete blood count or white blood cell differential analyses for absolute counts of neutrophils, bands, lymphocytes, monocytes, eosinophils and basophils (Table 1; *P*>0.05 for fVII-tPDT *vs* control by two-way ANOVA with Bonferroni post-test). Thus, we conclude that fVII-tPDT is effective and safe for the treatment of chemoresistant breast cancer *in vivo* in the mouse model.

## Discussion

Multidrug resistance is a common and major problem in the treatment of cancer, including breast cancer (Giai *et al*, 1991). In this paper we report the efficacy of fVII-tPDT using fVII-SnCe6 conjugate for the treatment of chemoresistant breast cancer by targeting TF on chemoresistant breast cancer cells and tumour VECs. In this study we show that fVII-tPDT eradicates chemoresistant breast cancer cells *in vitro* and significantly inhibits the tumour growth *in vivo* in a nude mouse model of chemoresistant breast cancer. To our knowledge, this is the first presentation of a novel, effective and dual neovascular- and cancer cell-targeting fVII-tPDT as an alternative to chemotherapy to bypass drug resistance of cancer.

Photodynamic therapy has several important advantages compared with chemotherapy. First, several studies have reported that MDR tumour lines were equally sensitive to PDT when compared with the chemosensitive parental lines (Kessel and Erickson, 1992; Kusuzaki *et al*, 2000). Second, the PDT procedure can be repeated multiple times if needed because there are no cumulative toxic effects and it is usually an outpatient procedure (Capella and Capella, 2003). Third, it can be used as a stand-alone modality or in combination with other therapies, including antiangiogenesis, surgery, radiotherapy and other treatments.

As discussed above, the main limitation of ntPDT is sensitisation to sunlight, such that patients undergoing PDT have to avoid direct sunlight and bright indoor light for a few days to weeks. To overcome the poor selectivity of PS, oestrogen receptors, epithelial growth receptors and HER-2 antigens expressed by breast cancer cells have been targeted by ligands or antibody/antibody fragments for the development of tPDT (Gijsens *et al*, 2000; Swamy *et al*, 2002, 2006; Savellano *et al*, 2005; Bhatti *et al*, 2008). In this regard, we were able to simultaneously target angiogenic VECs and cancer cells by targeting receptor TF using its natural ligand fVII-conjugated SnCe6 or verteporfin for the treatment of breast cancer in preclinical studies (Hu *et al*, 2010a, 2010b).

The reason for targeting TF by fVII was that TF is selectively expressed by angiogenic VECs and overexpressed by many types of cancer cells (Contrino *et al*, 1996; Hu *et al*, 1999; Hu and Garen, 2001; Tang *et al*, 2007). Moreover, fVII binds to TF with high affinity and specificity (Waxman *et al*, 1992). In the course of the development of the TF-targeting concept and therapeutic agents using fVII, Hu and Garen (2000, 2001) constructed the first TF-targeting agent, an fVII/IgG1 Fc immunoconjugate (called an Icon) for immunotherapy of cancer (Hu *et al*, 1999). Given that TF was detected on angiogenic VECs in cancer (Contrino *et al*, 1996; Hu *et al*, 1999; Hu and Garen, 2001; Tang *et al*, 2007; Cocco *et al*, 2010) and other pathological neovasculature-involved diseases such as wMD (Bora *et al*, 2003; Tezel *et al*, 2007) and endometriosis (Krikun *et al*, 2010), Icon immunotherapy was effective and safe for the treatment of cancer (Hu *et al*, 1999; Hu and Garen, 2000, 2001; Tang *et al*, 2007; Cocco *et al*, 2010), wMD (Bora *et al*, 2003; Tezel *et al*, 2007) and endometriosis (Krikun *et al*, 2010) in preclinical studies. In 2008, Shoji *et al* (2008) reported the use of an active site-inactivated recombinant human fVIIa (FFRck–fVIIa) as a carrier for the targeted delivery of a potent synthetic curcumin analogue (EF24) to TF-expressing tumour-associated VECs and tumours.

Here, we report a different TF-targeting therapeutics using fVII-conjugated photoactivatable SnCe6 for the treatment of chemoresistant human breast tumour. Contrino *et al* (1996) showed that TF was expressed on VECs of breast cancer cells but not on normal VECs of the breast from human subjects. We previously showed that mouse Icon (murine fVII/hIgG1 Fc) protein could selectively bind to tumour VECs in human melanoma and prostatic tumour xenografts but to VECs in several normal organs from severe combined immunodeficient mice (see Figure 3 in Hu *et al*, 1999 and Figure 7 in Hu and Garen, 2001, respectively), suggesting that TF is also selectively expressed by tumour VECs in human tumour xenografts but not by normal VECs from mice. However, it was previously unknown whether TF is expressed on VECs in chemoresistant breast tumours. Here, we showed for the first time that TF was indeed expressed on tumour VECs in chemoresistant MCF-7/MDR breast tumours. These results provide the basis and rationale for the efficacy of fVII-tPDT via targeting TF for the treatment of chemoresistant breast tumours. We then showed that fVII-tPDT induced both apoptosis and necrosis, as a mechanism of action, in MCF-7/MDR cancer cells and that fVII-tPDT was effective in eradicating MCF-7/MDR cancer cells *in vitro* and inhibiting tumour growth *in vivo*. Thus, fVII-tPDT as a localised therapy is different in the mechanism of action from Icon immunotherapy, which is a systemic modality and relies on host immune functions through NK cell- and complement-mediated cytotoxicity (Hu and Li, 2010). Taking the results together, we believe that fVII-tPDT can be used as a stand-alone modality and potentially in combination with Icon immunotherapy for the treatment of chemoresistant cancer.

In conclusion, we report here an effective and safe fVII-tPDT for the treatment of chemoresistant breast cancer by selectively targeting tumour VECs and the cancer cells. This TF-targeting fVII-tPDT could have therapeutic potential for the treatment of other chemoresistant cancers, as all tumours, including chemoresistant tumours and leukaemia, do need and have neovasculature (Bairey *et al*, 2000; Michaelis *et al*, 2009), in which TF is expressed by tumour VECs and/or cancer cells (Andoh *et al*, 1987; Bauer *et al*, 1989; Callander *et al*, 1992; Hair *et al*, 1996; Shoji *et al*, 1998; Hu *et al*, 1999; Hu and Garen, 2000, 2001).

## Figures and Tables

**Table 1:** CBC with WBC differential in the control and fVII-tPDT-treated mice

**CBC/differential**	**Control (*n*=5)**	**fVII-tPDT (*n*=5)**	***P-*value**	**Reference range**
Haemoglobin (g dl^–1^)	14.2±0.2	13.6±1.0	>0.05	13.0–15.0
Haematocrit (%)	45.3±2.0	42.6±3.3	>0.05	33.0–50.0
WBC (10^3^ *μ*l^–1^)	5.0±2.1	5.3±2.7	>0.05	5.0–10.0
RBC (10^6^ *μ*l^–1^)	9.4±0.2	8.7±0.9	>0.05	5.5–10.5
MCV (fl)	48.4±1.8	49.6±5.2	>0.05	NA
MCH (pg)	15.2±0.3	15.7±1.5	>0.05	NA
MCHC (g dl^–1^)	31.5±1.2	31.9±1.0	>0.05	NA
Platelet count (10^3^ *μ*l^–1^)	837.2±116.6	948.6±610.5	>0.05	NA
Neutrophils	1261.6±916.3	1788.0±1974.7	>0.05	NA
Neutrophilic bands	0.0±0.0	0.0±0.0	>0.05	NA
Lymphocytes	3581.6±1165.5	3373.2±718.0	>0.05	NA
Monocytes	60.4±30.7	54.6±83.4	>0.05	NA
Eosinophils	86.0±40.9	88.2±64.9	>0.05	NA
Basophils	50.4±21.2	16.0±22.8	>0.05	NA

Abbreviations: CBC=complete blood count; WBC=white blood cells; RBC=red blood cells; MCV=mean corpuscular volume (the average volume of the red cells, measured in femtolitres); MCH=mean corpuscular haemoglobin (the average amount of haemoglobin per red blood cell, in pg); MCHC=mean corpuscular haemoglobin concentration (the average concentration of haemoglobin in the cells); NA=not available from Antech Diagnostics Inc.; fVII-tPDT=factor VII-targeted photodynamic therapy.

*P*-values were analysed by two-way ANOVA using Prism 5.0c.
